# A growth-coupling strategy for improving the stability of terpenoid bioproduction in *Escherichia coli*

**DOI:** 10.1186/s12934-024-02548-1

**Published:** 2024-10-16

**Authors:** Jing Chong Tan, Qitiao Hu, Nigel S. Scrutton

**Affiliations:** Manchester Institute of Biotechnology, The University of Manchester, 131 Princess Street, Manchester, M1 7DN UK

**Keywords:** Industrial biomanufacturing, Population heterogeneity, Genetic stability, Phenotypic stability, Robustness, Evolution, Continuous culture, Plasmid loss, Metabolic engineering, Strain engineering

## Abstract

**Background:**

Achieving cost-competitiveness remains challenging for industrial biomanufacturing. With whole-cell biocatalysis, inefficiency presents when individual cells vary in their production levels. The problem exacerbates when the basis for such production heterogeneity is heritable. Here, evolution selects for the low- and non-producers, as they have lowered/abolished the cost of bioproduction to fitness. With the scale of population expansion required for industrial bioproduction, the asymmetrical enrichment can be severe enough to compromise the performance, and hence commercial viability of the bioprocess. Clearly, addressing production heterogeneity is crucial, especially in improving the stability of bioproduction across the cell generations. In this respect, we designed a growth-coupling strategy for terpenoid bioproduction in *Escherichia coli*. By knocking out the native 1-deoxy-D-xylulose 5-phosphate reductoisomerase (*dxr*) gene and introducing the heterologous mevalonate pathway, we created a chassis that relies solely on the latter for synthesis of all terpenoids. We hypothesise that the need to sustain the biosynthesis of endogenous life-sustaining terpenoids will impose a minimum level of productivity, which concomitantly improves the bioproduction of our target terpenoid.

**Results:**

Following the confirmation of lethality of a *dxr* knockout, we challenged the strains with a continuous plasmid-based bioproduction of linalool. The Δ*dxr* strain achieved an improved productivity profile in the first three days post-inoculation when compared to the parental strain. Productivity of the Δ*dxr* strain remained observable near the end of 12 days, and after a disruption in nutrient and oxygen supply in a separate run. Unlike the parental strain, the Δ*dxr* strain did not evolve the same deleterious mutations in the mevalonate pathway, nor a viable subgroup that had lost its resistance to the antibiotic selection pressure (a plausible plasmid loss event). We believe that this divergence in the evolution trajectories is indicative of a successful growth-coupling.

**Conclusion:**

We have demonstrated a proof of concept of a growth-coupling strategy that improves the performance, and stability of terpenoid bioproduction across cell generations. The strategy is relatively broad in scope, and easy to implement in the background as a ‘fail-safe’ against a fall in productivity below the imposed minimum. We thus believe this work will find widespread utility in our collective effort towards industrial bioproduction.

**Supplementary Information:**

The online version contains supplementary material available at 10.1186/s12934-024-02548-1.

## Background

**Fig. 1:**
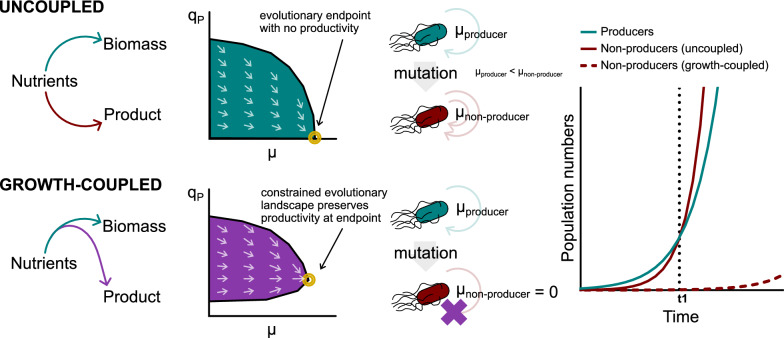
Growth-coupling controls production heterogeneity by restricting the evolutionary landscape. In an uncoupled strain, a trade-off is present between biomass growth and bioproduction for nutrient allocation. With natural selection, the overall direction of its evolutionary trajectory (arrows on teal graph) is towards the maximisation of specific growth rate (μ) at the expense of specific productivity (q_P_). In a simplified scenario where a producer (teal) mutates into a non-producer (dark red) with a specified probability, producers with a lower μ swiftly becomes dominated by non-producers (after time = t1 on the rightmost graph (solid lines), assuming no nutrient limitation). With growth-coupling, bioproduction is a necessary outcome when nutrients are used for biomass growth. This restricts the evolutionary landscape of the strain (purple graph); though the overall direction of its evolutionary trajectory is the same, productivity remains at the envisioned endpoint (gold concentric circles). Non-producers in the simplified scenario are no longer viable (purple cross), rendering the mutation event abortive to prevent their dominance (dashed dark red line of rightmost graph)

Industrial biotechnology has long been heralded as a possible means of supplanting the unsustainable petrochemical industry. However, progress has largely been restricted to the low-volume, high-margin businesses of the fine chemicals market, as cost-competitiveness remains an elusive goal [1]. With whole-cell biocatalysis, a key problem presents when individual cells vary in their level of production: inefficiencies result when low- or non-producers drain nutrients from the high-producers, to the detriment of the population’s overall performance in titres, rates, yields, and productivity [2–6]. Unfortunately, such production heterogeneity is inevitable. It can be attributed to the spontaneity of genetic mutations [4, 6], the stochastic noise of biological processes [2], and the responses to physicochemical fluctuations within the reactors [5, 6]. Often, the problem is further exacerbated when the basis for production heterogeneity is heritable by daughter cells. Here, evolution will preferentially select for the low- and non-producers, as the artificial imposition of bioproduction necessarily carries a cost to fitness. Rugbjerg et al. [4] demonstrated this with fourteen serial passages of a mevalonate producing *Escherichia coli* strain, wherein a decline in productivity to zero resulted from the increasing dominance of non-producing genetic mutants. Studies that have subjected bioproduction strains to serial passages often observe a similar productivity decline across generations [7, 8]. As populating the industrial-sized reactors will require such a degree of population expansion, it becomes apparent that the asymmetrical enrichment can significantly skew the makeup to favour the low- and non-producing variants (Fig. 1, uncoupled). The impaired performance of the bioprocess can be severe enough to compromise its commercial viability, thus manifesting as a scale-up failure [3, 6]. Continuous bioprocesses face even poorer prospects, as the possibility for population takeovers [9] by successively inferior variants allows for productivity to be effectively abolished in time. This is disappointing, as continuous processes can in principle lead to process intensification and simplification to create highly competitive bioproduction platforms [10, 11]. Clearly, limiting production heterogeneity is critical, especially if we also stabilise the inheritance of the bioproduction phenotype across the cell generations of the chassis.

In this respect, a promising approach is to engineer the dependence of growth and survivability on the cell’s individual level of production (i.e. coupling bioproduction to growth, or ‘growth-coupling’) [3, 12, 13]. Firstly, this reduces production heterogeneity by imposing a minimum level of productivity on each and every cell. Secondly, growth-coupling also stabilises the vertical inheritance of the bioproduction phenotype by restricting the chassis’ evolutionary landscape, as variants that fall below the imposed minimum cannot survive and propagate (Fig. 1, growth-coupled). These two factors thus collectively improve the overall performance of the bioprocess. Biological engineers that desired these outcomes have created various designs to effect growth-coupling [3]. The most direct approach rewires endogenous metabolism, to compel flux through the bioproduction pathway as a mandatory outcome if the cell were to survive and grow. For instance, Wang et al. [14] knocked out multiple pyruvate-yielding reactions in *E. coli*, creating a strain that must produce anthranilate alongside pyruvate if it were to survive on glycerol as the sole carbon source. Separately, Shen et al. [15] created an *E. coli* strain that must produce 1-butanol, to regenerate enough NAD^+^ to grow under anaerobic conditions. Such laboratory investigations into growth-coupling may turn to computational methods to probe strain designs [12, 13]. In this study, we looked to the evolutionary organisation of terpenoid biosynthetic pathways across the domains of life, for designing a strategy to couple growth with bioproduction of the same.

**Fig. 2:**
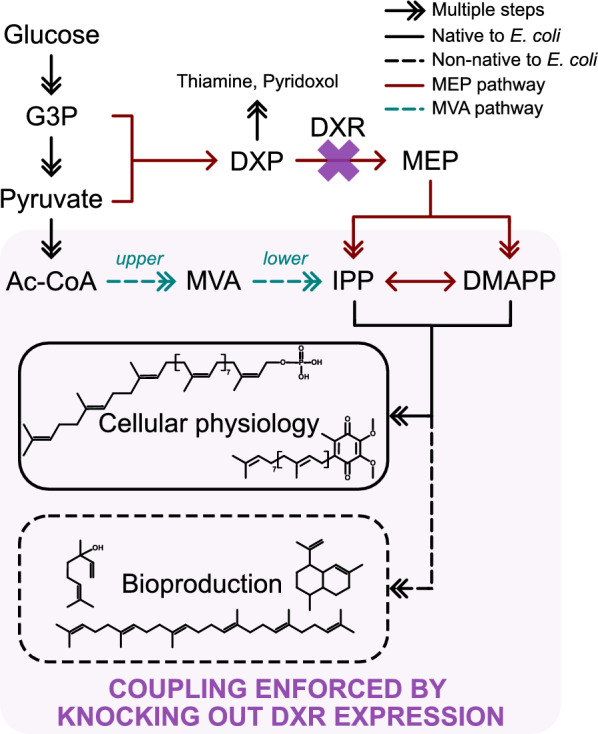
The convergence of the two canonical pathways for terpenoid synthesis in *E. coli*. In *E. coli*, terpenoid biosynthesis initiates from the condensation of the central metabolites glyceraldehyde-3-phosphate (G3P) and pyruvate to form 1-deoxy-D-xylulose 5-phosphate (DXP). As DXP can be channelled to other metabolic reactions, the conversion of DXP to the pathway’s namesake 2-C-methyl-D-erythritol 4-phosphate (MEP) by DXP reductoisomerase (DXR) is the rate-committing step. MEP is processed in five further steps to form the universal terpenoid precursors isopentenyl diphosphate (IPP) and dimethylallyl diphosphate (DMAPP). These precursors are condensed to form endogenous terpenoids involved in fundamental cellular physiology (solid black box), such as undecaprenyl phosphate (top) for cell wall synthesis, and ubiquinone-8 (bottom) for respiratory electron transfer. IPP and DMAPP are also responsible for bioproduction of industrially valuable terpenoids (dashed black box), such as linalool, amorphadiene and squalene (clockwise, starting from top left). In this respect, metabolic engineers may target the native MEP pathway (solid dark red lines), or co-opt the heterologous mevalonate (MVA) pathway (dashed teal lines) that occurs through the eponymous intermediate from acetyl-CoA (Ac-CoA). Steps upstream and downstream of MVA are conventionally labelled as the upper and lower MVA pathway respectively. Our growth-coupling strategy knocks out the native expression of DXR (purple cross), compelling the chassis to rely solely on the heterologous MVA pathway for synthesis of all terpenoids (purple box)

Terpenoids are diverse and ubiquitous natural compounds unified by having the five carbon isoprene as their building block. With broad utility across various industries in the capacity of both fine and commodity type chemicals, their potential commercial value has long since attracted efforts to develop microbial routes of production [16]. However, terpenoids are also involved in fundamental life-sustaining processes in all living cells, such as photosynthetic and respiratory electron transfer [17], prokaryotic peptidoglycan cell wall biosynthesis [18], localisation of eukaryotic membrane proteins [19], and (bacterio)chlorophyll biosynthesis in phototrophs [20]. Naturally, all free-living organisms retained the capacity to synthesise terpenoids, although perhaps relying on different metabolic pathways to do so. The 2-C-methyl-D-erythritol 4-phosphate (MEP) pathway and the mevalonate pathway are the two canonical biosynthetic routes: though differing in where they branch out from central metabolism and in the subsequent enzymes and metabolites involved, they both converge back onto the universal precursors isopentenyl diphosphate (IPP) and dimethylallyl diphosphate (DMAPP) (Fig. 2). Uniquely, the outcome of evolution has been that most bacteria rely solely on the MEP pathway for terpenoid biosynthesis, as is the case for the model organism *E. coli* [21, 22].

This sole dependence means that knocking out the first committed step of the MEP pathway (via *dxr*, refer to Fig. 2) in *E. coli* is lethal, and this has been demonstrated in the laboratory [23, 24]. However, growth can be rescued through the heterologous expression of the analogous mevalonate pathway, as biosynthesis of endogenous terpenoids through IPP and DMAPP is restored [24, 25]. Pfleger et al. [24] exploited this to create an *E. coli* whole-cell biosensor, whose growth was used for a high-throughput search for top mevalonate producers.

Here, we see this interchangeability of pathways as an opportunity for growth-coupling in *E. coli*. Engineering the mevalonate pathway in *E. coli* has generally found more success for terpenoid bioproduction [26, 27]. However, an evolutionary drive to inactivate the pathway remains [4, 28, 29], and this might arise from problems like an accumulation of cytotoxic intermediates [30, 31] and product [26, 32]. These issues relate to a suboptimal operation of the bioprocess, though addressing this is a Herculean task. Hence, we believe in the value of growth-coupling as a complementary strategy, whereby a Δ*dxr* chassis is utilised for engineering the mevalonate pathway. Here, the need to restore and sustain endogenous terpenoid biosynthesis will impose a minimum level of productive flux through the mevalonate pathway (Fig. 2, purple box). We hypothesise a resultant improvement in the performance of the bioprocess, notably through the improved stability in the bioproduction phenotype inherited across the cell generations of the chassis.

We evaluated this in the context of continuous plasmid-based bioproduction of linalool, a representative terpenoid with commercial applicability in the fuels [33], cosmetics [34], chemical manufacturing and food industries [35]. In our conditions tested, we observed the productivity of the Δ*dxr* strain to generally outperform the parental strain in the first three days post-inoculation. Stability of linalool bioproduction across cell generations did improve for the Δ*dxr* strain as well. Crucially, qualitative differences in the strains’ evolution suggests that these were the results of a successful growth-coupling.

## Methods

### Plasmids and cloning

CloneAmp HiFi (Takara Bio) was used for PCR amplification, and overlapping DNA segments were ligated with the In-Fusion kit (Takara Bio). Plasmids were maintained in DH5α (Table 1) by chemical transformation for cloning purposes. PCR, ligation, and transformation were conducted in accordance with the manufacturer’s instructions. Cells were propagated in LB (Luria–Bertani) broth at 37 °C with 100 μg/mL carbenicillin, 25 μg/mL chloramphenicol or 100 μg/mL streptomycin. Plasmid assemblies were verified with Sanger sequencing (Eurofins Genomics).

**Table 1:** *E. coli* strains used in the study

Strain	Genotype	Source
DH5α	*fhuA2::IS2* Δ(*mmuP-mhpD*)*169* Δ*phoA8 glnX44* ϕ80d[Δ*lacZ58*(*M15*)] *rfbD1 gyrA96 luxS11 recA1 endA1 rph*^WT^ *thiE1 hsdR17* [36]	NEB® 5-α, New England Biolabs
DH5α *dxr*FRT	DH5α with native *dxr* flanked by FRT	This work
DH5α Δ*dxr*	DH5α Δ*dxr*::FRT	This work

FRT: Flp recombinase target sequences

Plasmids (Table 2) constructed in this study were created by the homology-based ligation of two overlapping fragments, with both fragments previously generated by PCR amplification. For pTF-dxr, the first fragment was linearised pTF, and the second was a synthesised gene segment (Integrated DNA Technologies) containing the FRT-*dxr*-FRT donor DNA (dDNA) sequence. For pTet-Flp, the first fragment contained the gene encoding the Flp recombinase from pCP20, and the second was the backbone of pTet-RFP (i.e. excluding the mRFP1 gene and T_rrnBT1_). For pLMVA-Lin, the first fragment contained the lower mevalonate pathway and the GPPS-LinS operons from pMVA-Lin, and the second contained the p15A origin, Amp^R^ and LacI sequences from pMVA-Lin as well. Here, the primers were designed in a manner that upon fusion of the fragments to form pLMVA-Lin, the T_rrnBT1-T7Te_ terminator of the GPPS-LinS operon would be replaced with T_ECK120015170_ [37]. Aside from this change, pLMVA-Lin is essentially pMVA-Lin with the upper mevalonate pathway operon removed.

**Table 2:** Plasmids used in the study

Plasmid	Description	Source
pSIMcpf1	pSC101^T^^S^ ori; Hyg^R^; P_R_: cI^TS^; P_L_: Gam-Beta-Exo; P_BAD_: AraC, crRNA_pMB1_-T_BBa_B1006_; P_JS23151_: AsCpf1-T_BBa_B1002_	[38]
pTF	pMB1 ori; Stm^R^; P_JS23119_	[38]
pTF-dxr	pMB1 ori; Stm^R^; P_JS23119_: crRNA_dxr_-T_rrnBT1-T7Te_; dDNA_FRT-dxr-FRT_	This work
pCP20	pSC101^T^^S^ ori; Amp^R^, Cam^R^; P_R_: cI^TS^, Flp	[39]
pTet-RFP	pSC101 ori; Cam^R^; P_tet_: TetR, mRFP1-T_rrnBT1-T7Te_	pBbS2c-RFP [40]
pTet-Flp	pSC101 ori; Cam^R^; P_tet_: TetR, Flp-T_T7Te_	This work
pTrc-RFP	p15A ori; Amp^R^; P_lacIq_: LacI; P_trc_: mRFP1-T_rrnBT1-T7Te_	pBbA1a-RFP [40]
pMVA-Lin	p15A ori; Amp^R^; P_lacI_: LacI; P_lacUV5_: AACT-HMGS-HMGR; P_J23116_: MK-PMK-PMD-IDI-T_BBa_B1002_; P_trc_: GPPS-LinS-T_rrnBT1-T7Te_	pMVA-GLinS NR2 [28]
pLMVA-Lin	p15A ori; Amp^R^; P_lacI_: LacI; P_J23116_: MK-PMK-PMD-IDI-T_BBa_B1002_; P_trc_: GPPS-LinS-T_ECK120015170_	This work

P and T: promoters and terminators respectively

^TS^: temperature-sensitive; FRT: Flp recombinase target sequences; Amp^R^, Cam^R^, Stm^R^, Hyg^R^: enzyme for resistance to ampicillin/carbenicillin, chloramphenicol, streptomycin and hygromycin respectively; crRNA: CRISPR RNA array recognised by the AsCpf1 Cas12a; dDNA: donor DNA used for repair

Refer to Figure S2 for plasmid maps and full gene names

### Genome editing to obtain DH5α *dxr*FRT

To obtain DH5α *dxr*FRT (Table 1), we followed the protocol for a CRISPR-Cas12a system [38] to edit the genome of DH5α. Specifically, we directed Cas12a to cleave within the intergenic segments upstream and downstream of the native *dxr* gene. The dDNA used for repair was designed so that the replaced *dxr* gene would be flanked by a set of Flp recombinase target (FRT) sequences. Both CRISPR plasmids (pTF-dxr and pSIMcpf1) were cured as per the published protocol.

Following the edit, the *dxr* locus was PCR amplified by a set of flanking primers, and the amplicons were gel-purified and extracted with the Monarch DNA Gel Extraction Kit (New England Biolabs). The edits were verified with Sanger sequencing of these amplicons.

### Growth assays for investigating the lethality of a *dxr* knockout

DH5α *dxr*FRT was electroporated with (1) pTet-RFP and pLMVA-Lin (‘no knock out control’), (2) pTet-Flp and pTrc-RFP (‘no rescue control’) and (3) pTet-Flp and pLMVA-Lin (‘knock out + rescue’). Eight single colonies were picked for each of the three strains. Each colony was transferred to 1 mL cultures of (i) base medium (phosphate-buffered Terrific Broth (Formedium) supplemented with 0.4% D-glucose, 100 μg/mL carbenicillin and 25 μg/mL chloramphenicol), (ii) base medium supplemented with 200 nM anhydrotetracycline and (iii) base medium supplemented with 200 nM anhydrotetracycline and 1 mM (R)-mevalonate. (R)-mevalonate was prepared from hydrolysing (R)-mevalonolactone (Sigma-Aldrich) with 1 M KOH at 37 °C for 30 min [41]. Each 1 mL culture was housed within a well of a 24-well plate. To reduce the loss of culture volume, the plate was covered with its lid and sealed with cling film. The cultures were grown with shaking for 19 h at 37 °C. Aliquots were transferred to a 96-well plate, for quantitation of optical density at 600 nm (OD_600_) with a FLUOstar Omega microplate reader (BMG LABTECH). Statistical significance was evaluated for the effect of anhydrotetracycline induction (conditions (i) vs (ii)), and the influence of mevalonate on this (conditions (ii) vs (iii)). This was a Wilcoxon matched-pairs signed rank test conducted on GraphPad Prism 9.3.1.

To determine the knockout status of the *dxr* locus, suspensions of the cultures at the end of the assay were analysed by colony PCR with GoTaq G2 Green (Promega). Amplicons were visualised by agarose gel electrophoresis.

Cultures of the ‘knock out + rescue’ strain (grown with anhydrotetracycline and mevalonate) recovered at the end of the 19 h growth assay were streaked onto LB plates. These plates were supplemented with 100 μg/mL carbenicillin, 25 μg/mL chloramphenicol, 200 nM anhydrotetracycline and 1 mM (R)-mevalonate. Colonies that grew following 37 °C incubation were checked for the status of their *dxr* locus by the colony PCR analysis detailed above. Eleven pure colonies with the *dxr* knockout genotype (DH5α Δ*dxr*) were recovered and subjected to the same growth analysis above. With the base medium of phosphate-buffered Terrific Broth maintained at 30 °C supplemented with 0.4% D-glucose and 100 μg/mL carbenicillin, only two conditions were tested this time: with and without 1 mM (R)-mevalonate supplementation. The culture was maintained at 37 °C with shaking, and OD600 was measured across five days.

### Continuous linalool bioproduction

DH5α *dxr*FRT was electroporated with pTet-Flp and pMVA-Lin. The knockout of the *dxr* gene was induced in 1 mL cultures as described above (condition (iii)). Here, (R)-mevalonate was substituted with 6.25 μM of IPTG for rescue of endogenous terpenoid biosynthesis. Following overnight growth, pure colonies were isolated by streaking on plates, and genomic DNA was extracted from them with the PureLink Genomic DNA Mini Kit (Invitrogen). The same PCR verification was conducted on the genomic DNA to verify the status of the *dxr* locus. Colonies with the *dxr* gene knocked out (DH5α Δ*dxr*) were then evaluated in a continuous bioproduction of linalool – these are referred to as the ‘Δ*dxr*’ strain. Their performance was benchmarked against the ‘parental’ strain, the latter being the unedited DH5α that had been chemically transformed with the same pMVA-Lin. For both strains, overnight cultures that were uninduced (parental strain) or induced with 6.25 μM of IPTG for rescue (Δ*dxr* strain) were used to prepare cryostocks.

At the start of each run, plate cultures of both strains were freshly prepared by streaking from cryostock. Single colonies were inoculated into 50 mL of phosphate-buffered Terrific Broth, supplemented with 0.4% D-glucose and 100 μg/mL carbenicillin. 6.25 μM of IPTG was added only for the Δ*dxr* strain. The cultures were grown overnight with shaking at 37 °C (Δ*dxr* strain) and 30 °C (parental strain). The lower temperature of the latter was thought to minimise any permanent loss of productivity at this stage. These seed cultures were used to separately inoculate a 1 L Multifors bioreactor (INFORS HT) at a starting OD600 of approximately 0.1. The fresh medium in the reactors was 400 mL of phosphate-buffered Terrific Broth maintained at 30 °C, supplemented with 0.4% D-glucose, 100 μg/mL carbenicillin and 50 μM of IPTG. This is the optimal IPTG concentration established for this pathway [28]. To account for the different lag phases between the two strains, the switch from batch to continuous mode was arbitrarily set at the point when their respective pH rebounds from its initial decline. The feed composition was the same as the batch medium, and was pumped into the reactors at a constant flow rate specified for that experiment. The volume of the culture was maintained by pumping excess culture out from a pipe affixed at a specified height. Peristaltic pumps (Masterflex L/S) were used to drive the inflow and outflow. The specific growth rate imposed at steady-state for a specified flow rate can be calculated by Eq. 1. Throughout the run, pH was controlled at the set point of 7 with 2 M sulfuric acid and 25% aqueous ammonia. The set point for dissolved oxygen was 30% and controlled by manipulating the stirring rate between 300 and 1200 rpm. Polypropylene glycol P2000 was manually added to reduce foaming when needed.

1 $$\text{Specific Growth Rate at Steady State}=\text{Dilution Rate}= \frac{\text{Flow Rate}}{\text{Culture Volume}}$$

Across the run at each time point, one sample was drawn from each reactor (parental versus Δ*dxr*) for the following analyses:

1. Two aliquots were drawn from each sample to measure OD600 with the Cary 60 UV–Vis Spectrophotometer (Agilent Technologies). OD600 values were reported as an average of these technical duplicates.
2. Two aliquots were drawn from each sample and extracted in parallel for linalool. Here, an equal volume of ethyl acetate spiked with 0.005% sec-butylbenzene was added to the aliquots. The organic phase was dried with anhydrous magnesium sulfate, and processed with a 7890B/5977A Gas-Chromatograph/Mass Selective Detector (Agilent Technologies). Separation was conducted through a DB-WAX column (30 m length × 0.32 mm inner diameter, 0.25 µm film thickness, Agilent Technologies). The injector temperature was set at 240 °C with a split ratio of 20:1. 1 µL of each sample was injected. The carrier gas was helium with a flow rate of 2 mL/min and a pressure of 4.6 psi. The oven program used is as follows: 50 °C (1 min hold), ramp to 68 °C at 5 °C/min (2 min hold), and ramp to 230 °C at 25 °C/min (2 min hold). The ion source temperature of the mass spectrometer was set to 230 °C, and spectra were recorded from m/z 50 to m/z 250. Linalool peaks were verified by referencing the NIST database of MS spectra and fragmentation patterns. Linalool titres were quantitated by comparing the peak area ratios (relative to the sec-butylbenzene internal standard) with those generated from a standard curve. Linalool titres were reported as an average of these technical duplicates. The linalool standard curve was prepared from 0.79 mg/L to 113.8 mg/L with a chemical standard purchased from Sigma-Aldrich.
3. With normalisation to OD600, an aliquot was drawn from each sample for plasmid extraction with the E.Z.N.A® Plasmid DNA Mini Kit (Omega Bio-tek). Whole plasmid sequencing was performed by Plasmidsaurus using Oxford Nanopore Technology with custom analysis and annotation. For visualisation of plasmid on an agarose gel, plasmids were first digested with *Nco*I-HF (New England Biolabs) as per the manufacturer’s instructions.
4. In the third run, colony forming units (CFU) were counted on the first day post-inoculation. Two aliquots were drawn from each sample for parallel processing through a ten-fold serial dilution. Dilutions were each spread onto two sets of plates; one made to match the composition of the fresh medium used for the continuous bioproduction, and the other the same but without carbenicillin. Plates were grown at 30 °C. CFU counts were reported as an average of these technical duplicates. Statistical significance was evaluated for the effect of carbenicillin supplementation, with an assumption that the CFU counts approximated a log-normal distribution [42]. This was a paired t-test conducted on the log transformed values in GraphPad Prism 9.3.1.

## Results

### Knocking out *dxr *was lethal for *E. coli*, but can be rescued by mevalonate pathway expression

**Fig. 3:**
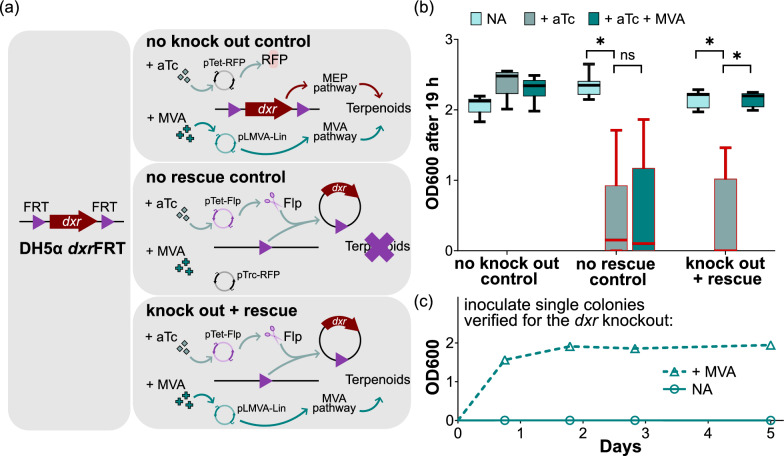
Growth arrest from knocking out *dxr* was rescued by the lower mevalonate pathway. **a** The genomic *dxr* gene of the DH5α *dxr*FRT strain was flanked with Flp recombinase target (FRT) sequences (left box). DH5α *dxr*FRT was transformed with varying plasmid combinations to yield the three strains on the right boxes. We depict here the impact of the plasmids on terpenoid biosynthesis, upon supplementation with anhydrotetracycline (aTc) and mevalonate (MVA). aTc induced expression of the Flp recombinase from pTet-Flp, which knocked out the *dxr* gene to inactivate the native 2-C-methyl-D-erythritol 4-phosphate (MEP) pathway. Mevalonate can rescue terpenoid biosynthesis through the heterologous lower MVA pathway, which was constitutively expressed by pLMVA-Lin. pTet-RFP and pTrc-RFP served as the respective controls. **b** Strains in **a** were grown for 19 h with varying culture supplementations, and OD600 was recorded at the end. Eight biological replicates were tested per strain. For p-values of pairwise comparisons, * indicates < 0.05, and ‘ns’ indicates > 0.05. Where knockout was effected without rescue (boxes outlined in red), analysis by colony PCR (Figure S1a) revealed that knockout was imperfect. Growth from these escapees was responsible for the wide spread of the data around the median. **c** To unequivocally demonstrate the lethality of the *dxr* knockout, we isolated single colonies verified for this genotype. Eleven were evaluated for their ability to grow with and without MVA supplementation. Error bars that represent the standard deviation of these eleven biological replicates were smaller than the displayed symbols

Utilising a CRISPR-Cas12a genome editor [38], we flanked the *dxr* locus of *E. coli* DH5α with a set of Flp recombinase target (FRT) sequences, creating the strain DH5α *dxr*FRT. This strain was transformed with pTet-Flp, and either pTrc-RFP (‘no rescue control’, Fig. 3) or pLMVA-Lin (‘knock out + rescue’, Fig. 3), and allowed to grow for 19 h. If anhydrotetracycline was added to express the Flp recombinase, the genomic *dxr* gene would be knocked out by recombination across the flanking FRT sequences. Here, a resultant growth arrest was demonstrated in both strains (Fig. 3b, respective middle boxes). If mevalonate was also added to the culture, growth arrest was circumvented only for the strain with pLMVA-Lin (Fig. 3b, respective right boxes), which constitutively expresses the lower segment of the mevalonate pathway. Growth arrest was not observed under all conditions for the ‘no knock out control’, which had been transformed with pTet-RFP and pLMVA-Lin.

However, where growth arrests were observed when comparing between the average OD600 values, they were sporadic amidst the biological replicates to cause high variability in the data. Colony PCR revealed that the aberrant escapees were composed of either a mixed population of cells with and without the *dxr* gene knocked out, or a pure population of the latter (Figure S1a). The mixed cultures generated from the pTet-Flp and pLMVA-Lin combination were thus streaked out to isolate pure individual colonies. Selected colonies were verified for the knockout (Figure S1b), and then subjected to the same growth arrest/rescue assay. This time, a complete arrest was observed in all biological replicates for up to 5 days, and this was also rescuable by mevalonate supplementation (Fig. 3c).

### The Δ*dxr* strain achieved higher peak titres, and maintained productivity for longer

**Fig. 4:**
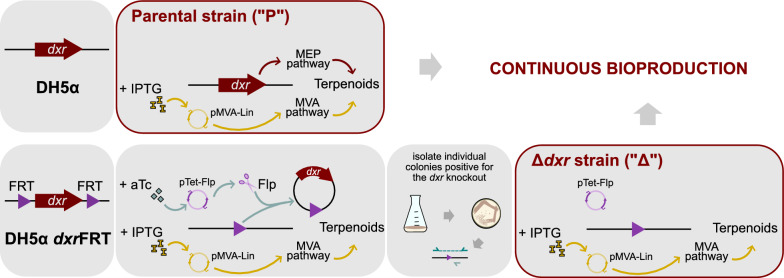
Generating the strains for continuous bioproduction of linalool. To generate the parental strain, the DH5α strain was transformed with pMVA-Lin. Terpenoid synthesis can occur either through the native 2-C-methyl-D-erythritol 4-phosphate (MEP) pathway via *dxr*, or through the heterologous mevalonate (MVA) pathway with IPTG induction. To generate the Δ*dxr* strain, DH5α *dxr*FRT was first transformed with pTet-Flp and pMVA-Lin. Anhydrotetracycline (aTc) induced the expression of the Flp recombinase from pTet-Flp, which knocked out the *dxr* gene. Terpenoid synthesis was restored by inducing the heterologous MVA pathway on pMVA-Lin with IPTG. The Δ*dxr* strain was obtained by isolating pure colonies with a verified knockout. This strain relies only on the said MVA pathway for terpenoid synthesis

We transformed DH5α *dxr*FRT with pTet-Flp and pMVA-Lin, induced both plasmids, and isolated a pure knockout strain (Fig. 4). PCR analysis was conducted on extracted genomic DNA to confirm the deletion of the *dxr* gene (Figure S1c). This strain is henceforth referred to as the ‘Δ*dxr*’ strain. We then investigated the effects of the knockout on a continuous linalool bioproduction process encoded by the pMVA-Lin plasmid. Its performance was benchmarked against the unedited ‘parental’ strain, which had been transformed with the same pMVA-Lin plasmid.

**Fig. 5:**
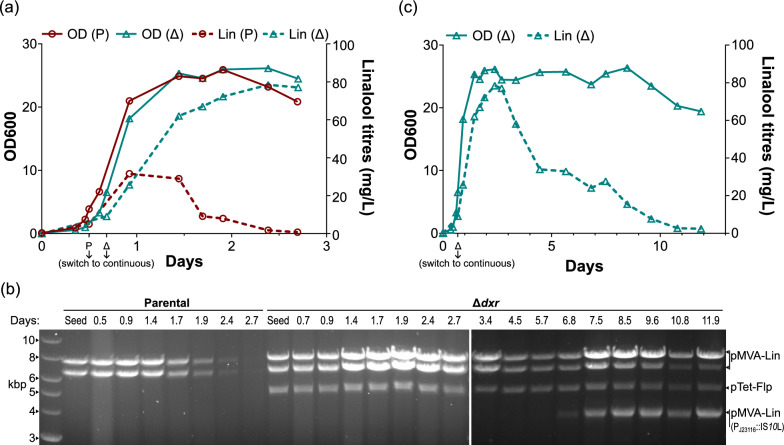
(Run 1) Continuous linalool bioproduction at μ = 0.15 h^−1^. **a** OD600 and linalool titres measured for the first three days post-inoculation for the parental (P) and Δ*dxr* strain (Δ). Error bars that represent the standard deviation of the technical duplicates were smaller than the displayed symbols. **b** At the respective time points (top row), plasmids were normalised to OD600 for extraction, and then digested with *Nco*I. The DNA ladder is on the leftmost lane. ‘Seed’ refers to the respective seed cultures used for inoculating the reactors. Assignment of the bands to the respective plasmids are indicated on the right. ‘pMVA-Lin (P_J23116_::IS*10*L)’ refers to the IS*10*-left inserted pMVA-Lin. The bands for the time points after the third day were analysed on a separate gel, and the two images are aligned by their DNA ladders. Refer to Figure S2 for plasmid maps. **c** OD600 and linalool titres measured from the Δ*dxr* strain for the full 12 days. Error bars that represent the standard deviation of the technical duplicates were smaller than the displayed symbols

In this first run (Run 1), we fixed a dilution rate that imposed a specific growth rate at about 0.15 h^−1^. In the first three days, the OD600 of the Δ*dxr* strain demonstrated a similar trajectory to the parental strain, albeit with a slightly longer lag phase (Fig. 5a, solid lines). However, the Δ*dxr* strain achieved a higher peak titre of 79 mg/L, relative to the parental strain’s at 32 mg/L (Fig. 5a, dashed lines). Only the parental strain recorded a decline in titres within these three days. This was concomitant with a decrease in band intensities, when the pMVA-Lin plasmid was extracted from the population and analysed on a gel (Fig. 5b).

Owing to excessive foaming, we were unable to maintain the parental strain’s culture beyond the third day. This was not an issue for the Δ*dxr* strain, though a productivity decline was observed across the additional nine days (Fig. 5c). At the end of the run, sequencing of whole plasmids extracted from the population revealed the appearance of a new species. This was characterised by an IS*10*-left (IS*10*L) insertion into the constitutive P_J23116_ promoter of the lower mevalonate pathway, to yield the plasmid labelled ‘pMVA-Lin (P_J23116_::IS*10*L)’ (Figure S2d). As the insertion introduces additional *Nco*I cut sites, the net change in the band pattern is the loss of the 6.5 kbp band, and the gain of two approximate 3.8 kbp bands that overlapped with each other. Both the gel analysis (Fig. 5b) and the frequency distribution of the read lengths (Figure S3a) suggests that the final population was a mix of those possessing pMVA-Lin and pMVA-Lin (P_J23116_::IS*10*L). The IS*10*L element can be mapped onto positions 168,787–170,113 and 261,651–262,977 of the genome of our chassis strain (NEB5α; sense strand of the Genbank entry [36]).

### In contrast to an IS*10*-left insertion, linalool bioproduction was abolished in the parental strain through extensive deletions of the plasmid

**Fig. 6:**
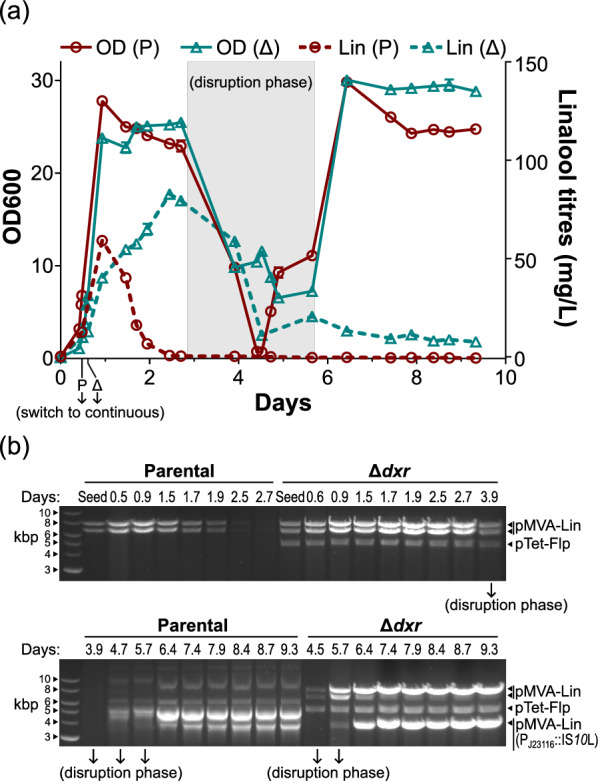
(Run 2) Continuous linalool bioproduction at μ = 0.15 h^−1^, with an intervening disruption. **a** OD600 and linalool titres measured for the parental (P) and Δ*dxr* strain (Δ). Compressed air supply was cut off throughout the entire disruption phase, which overlaps with a halt in fresh nutrient influx for the first 16 h. Error bars represent the standard deviation of the technical duplicates, though they may be smaller than the displayed symbols. **b** At the respective time points (top row), plasmids were normalised to OD600 for extraction, and then digested with *Nco*I. The DNA ladder is on the leftmost lane. ‘Seed’ refers to the respective seed cultures used for inoculating the reactors. Assignment of the bands to the respective plasmids are indicated on the right. ‘pMVA-Lin (P_J23116_::IS*10*L)’ refers to the IS*10*-left inserted pMVA-Lin. The two uncut mutant plasmids of the parental strain produced the five-band pattern observed. Refer to Figure S2 for plasmid maps

We repeated the same conditions to set the specific growth rate at about 0.15 h^−1^ again (Run 2). The overall trends in the first three days were the same as Run 1: the parental strain achieved a lower peak titre, and the rapid decline in productivity thereafter matched a decrease in plasmid band intensities (Fig. 6a).

Additionally, this run saw a three-day disruption that involved a loss of compressed air supply throughout. In the first 16 h of the disruption, this had also overlapped with the cessation of the influx of fresh feed, and hence of any culture dilution and turnover (i.e. a temporary batch process). This abrupt decrease in oxygen and nutrient supply presented a physiological challenge to the strains, loosely mimicking the concentration gradients that cells endure within large bioreactors. Here, we observed an expected decline in OD600, though both strains eventually made a full recovery. The effect on productivity however, was drastically different. For the parental strain, the progressive loss of productivity occurring even before the disruption could not be reversed at all. An abnormal plasmid band pattern appeared during the disruption, and enriched within the population during the recovery phase (Fig. 6b). Whole plasmid sequencing at the end revealed two homologous-recombinant mutants, both with large swathes of intervening genes deleted to render them completely production-defective (Figure S2c). As the deletions removed all *Nco*I cut sites, the five-band pattern observed should represent the various structural isoforms of the two uncut plasmids. They were dominating the bimodal distribution of the read lengths, suggesting that there was no intact pMVA-Lin present within the population (Figure S3b). In contrast, the decline in productivity of the Δ*dxr* strain eventually stopped to maintain titres at about 10 mg/L (Fig. 6a). We saw the appearance of the same pMVA-Lin (P_J23116_::IS*10*L) mutant plasmid described in Run 1. Although the gel analysis suggests that the final population was still a mix (Fig. 6b), the balance was likely further tilted towards the IS*10*L variant. This was corroborated by the frequency distribution of the sequencing read lengths, which shows the counts of pMVA-Lin to only be near background levels (Figure S3b).

### The Δ*dxr* strain could tolerate more challenging bioprocess conditions

**Fig. 7:**
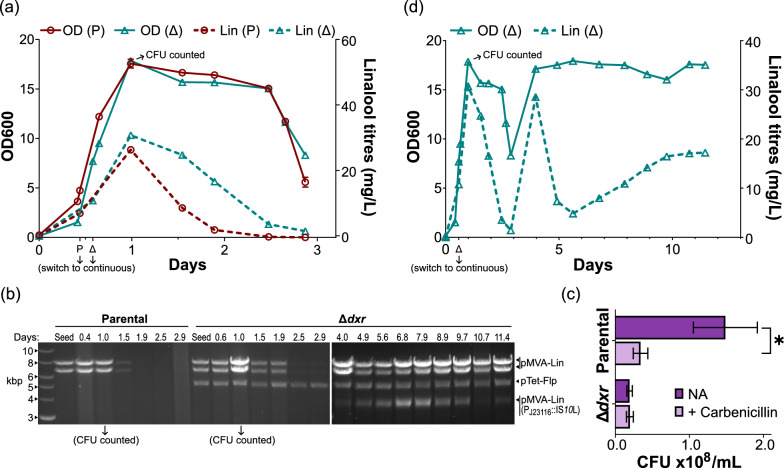
(Run 3) Continuous linalool bioproduction at μ = 0.2 h^−1^. **a** OD600 and linalool titres measured for the first three days post-inoculation for the parental (P) and Δ*dxr* strain (Δ). Error bars represent the standard deviation of the technical duplicates, though they may be smaller than the displayed symbols. **b** At the respective time points (top row), plasmids were normalised to OD600 for extraction, and then digested with *Nco*I. The DNA ladder is on the leftmost lane. ‘Seed’ refers to the respective seed cultures used for inoculating the reactors. Assignment of the bands to the respective plasmids are indicated on the right. ‘pMVA-Lin (P_J23116_::IS*10*L)’ refers to the IS*10*-left inserted pMVA-Lin. The bands for the time points after the third day were analysed on a separate gel, and the two images are aligned by their DNA ladders. Refer to Figure S2 for plasmid maps. **c** Colony forming units (CFU) counts at 1 day post-inoculation. Error bars represent the standard deviation of the technical duplicates. * indicates a pairwise comparison of the log-transformed values that has p-value < 0.05. **d** OD600 and linalool titres measured from the Δ*dxr* strain for the full 12 days. Error bars that represent the standard deviation of the technical duplicates were smaller than the displayed symbols

We pushed the cells to maintain a higher specific growth rate at about 0.2 h^−1^ by increasing the flow rate of fresh medium influx (Run 3). Here, we observed the Δ*dxr* strain to closely match the parental strain in both OD600 and productivity (Fig. 7a). There was a notable decrease in OD600 starting 2.5 days post-inoculation, although productivity was already declining from the first day. The latter’s decline appeared more pronounced for the parental strain, and both were matched with a corresponding decrease in band intensities for pMVA-Lin (Fig. 7b). To evaluate the extent of heterogeneity at peak productivity, we plated an aliquot of each culture on agar supplemented with and without carbenicillin (the antibiotic marker for pMVA-Lin). Only the parental strain produced a decrease in colony forming unit (CFU) counts in the presence of carbenicillin (Fig. 7c).

Once again, we were unable to maintain the parental strain’s culture for longer due to excessive foaming. For the Δ*dxr* strain, productivity recovered alongside OD600 by the fourth day (Fig. 7d). Productivity was always present thereafter, albeit fluctuating independently of OD600. This was concurrent with the emergence of the 3.8 kbp band in the population, which is suggestive of the same pMVA-Lin (P_J23116_::IS*10*L) mutant plasmid again (Fig. 7b).

## Discussion

This study has examined the feasibility of using a Δ*dxr* (MEP pathway-deficient) *E. coli* chassis, for coupling the bioproduction of linalool to growth.

We first replicated the lethality of a *dxr* knockout by demonstrating a complete growth arrest for up to 5 days. This could be avoided with mevalonate supplementation, but was contingent on the presence of pLMVA-Lin. As mevalonate is non-native to *E. coli*, the rescue can only be attributed to its action through the encoded lower mevalonate pathway, thus linking growth to IPP/DMAPP biosynthesis. This contextual growth requirement underpins our growth-coupling strategy: when simultaneously using the mevalonate pathway for producing a target terpenoid, the need to synthesise endogenous life-sustaining terpenoids will impose a minimum level of productivity for bioproduction as well. There will also be a restriction in the strain’s evolutionary landscape, as any variants of inadequate IPP/DMAPP synthesis cannot proliferate to dominate the population.

We evaluated the strategy in the context of continuous bioproduction of linalool. The Δ*dxr* strain possessed the complete mevalonate pathway encoded by pMVA-Lin, and its constant induction obviated the need for mevalonate supplementation. Continuous turnover of culture forces the population to maintain a minimum specific growth rate, and creates an environment of successive population takeovers by any mutants that evolved a higher relative fitness [9]. Titres of linalool measured at each time point represents the balance between bioproduction, and depletion from dilution and volatilisation. Hence, we were able to infer linalool productivity from the trends in the titres measured over time. Collectively, this setup compels the chassis to continuously replicate and evolve over the entire course of each run, thus laying bare the stability of bioproduction across its successive generations.

### Divergence in the type of mutants evolved suggests a successful growth-coupling

In all three runs, we were fascinated by the repeated enrichment of the same pMVA-Lin (P_J23116_::IS*10*L) mutant plasmid within the Δ*dxr* population. Hypothetically, mutants that have evolved to abolish bioproduction would have the greatest improvement in fitness, as resources for biomass formation no longer need to be partitioned away. Considering that the population of a continuous culture is repeatedly taken over by successively fitter variants, it is thus intriguing that the Δ*dxr* strain did not evolve a mutant that had unequivocally abolished bioproduction. Firstly, it did not yield the same recombinant mutants that had evolved from the parental strain in Run 2, despite having gone through the same bioprocess conditions. Secondly, the evolution of our transposon mutant with a mere promoter disruption is striking, when contrasted against two, far more deleterious possibilities. For instance, Rugbjerg et al. [4] demonstrated that both IS*10* and IS*186* (also mapped onto the NEB5α genome [36]) can insert into the first gene of our upper mevalonate pathway (AACT). Alternatively, IS*10* can insert into the first gene of our lower mevalonate pathway (MK) via two consensus target sequences [43, 44]. Both of these evolutionary pathways were disregarded in our Δ*dxr* strain, in favour of the P_J23116_ insertion via a non-consensus sequence. We believe that these observations point to an inability of the strain to abolish bioproduction, and a constrained evolutionary landscape that resulted from our success in growth-coupling.

A question that follows here is the impact and significance of our IS*10*L mutation. Firstly, the persistence of linalool detected in the Δ*dxr* culture implies a maintenance of productivity, in order to counter any product loss from volatilisation and dilution. Hence, we can be certain that the population is still expressing the mevalonate pathway at the end of each run. In this respect, our IS*10*L insertion may have retained some expression of the lower mevalonate pathway, as studies have shown that transposition can result in the expression of adjacent genes [45, 46]. In the case of IS*10*, most studies implicate a probable transcriptional read-through initiating from within the mobile element itself [47–51]. The orientation of IS*10*L in our study suggests that read-through may initiate from either P_III_ or P_OUTIIp_, and both promoters have previously been shown to be transcriptionally active in *E. coli* ([52] and [53] respectively). Although they used the IS*10*-Right (IS*10*R) variant for their study, we have verified that the segment containing both promoters (nucleotides 1000–1230 of IS*10*) is the same between IS*10*L and IS*10*R (refer to the NEB5α genome map [36], and to Fig. 5 of [54]). This specific IS*10*L mutant of ours may thus represent an evolutionary compromise to the growth-coupling; not a complete pathway inactivation, but a reduction in expression to levels just sufficient for the population to sustain endogenous terpenoid biosynthesis.

### Divergence in the evolution of carbenicillin susceptibility suggests a successful growth-coupling

On the first day post-inoculation of the third run, we discovered that bulk of the parental population had already evolved a susceptibility to carbenicillin, the antibiotic marker for pMVA-Lin. Contrary to this, the Δ*dxr* population remained uniformly resistant. As we had applied the same carbenicillin regime to both continuous cultures, these contrasting observations can only be attributed to the *dxr* genotype – the sole difference between the two strains. We believe that this evolutionary divergence of carbenicillin sensitivity is yet another indication of a successful growth-coupling: with the two phenotypes genetically linked on pMVA-Lin, the maintenance of resistance in the Δ*dxr* strain resulted as a knock-on effect from the selection pressure to sustain terpenoid biosynthesis.

Plasmid loss features repeatedly behind failures in bioproduction [55–57], and the correlation observed between resistance and bioproduction here does put forward its role as an underlying mechanism. Firstly, stochastic or regulatory [58, 59] failure in plasmid partitioning is commonplace, such that plasmid-free mutants can often be recovered from an expansion of plasmid-bearing progenitors [60, 61]. This subgroup can persist despite antibiotic selection, as resistance exists as a cooperative trait at the population level [62–67]. Beta-lactams (like carbenicillin) suffers notoriously from this, as extracellular dissemination [62, 63] of beta-lactamases (e.g. through outer membrane vesicles [68–70]) accelerates the detoxification of the bulk environment for all. In our case, it is evident that the rate of carbenicillin replenishment in the feed was insufficient to combat this [71]. With a resistant subgroup persisting to maintain detoxification, the loss of pMVA-Lin from the rest would concur with the decrease in band intensities and productivity observed for the parental population. This conditional abolishment of bioproduction may represent an intermediate state in evolution, lasting until the emergence of a mechanism to uncouple bioproduction from resistance (e.g. the recombinant mutants in Run 2). This would not have been possible for a growth-coupled strain, as a plasmid-free mutant would not be able to rescue terpenoid biosynthesis. This would explain why we could only recover resistant variants from the Δ*dxr* population.

### The confounding influence of bioprocess conditions

The conditions chosen for a continuous bioprocess profoundly influences the population’s response to the constant turnover of culture. The population may acclimatise after a temporary decline [72, 73], or even undergo an oscillatory cycle between washout and recovery [74, 75]. Crucially, these dynamics are accompanied by complex changes in morphology, gene expression and metabolism [73–75], which necessarily impacts productivity as well. We demonstrated the significance of the flow rate choice here, with the impaired performance of the Δ*dxr* strain in its third run vis-à-vis its first and second run. The population may have been unable to accommodate the higher specific growth rate imposed by the higher flow rate, resulting in the initial dip in both OD600 and linalool titres in the first three days. Similarly, we could only postulate about the decrease in band intensities as a sign of plasmid loss, as the concurrent reduction in specific growth rates observed may influence plasmid copy numbers as well [76].

A challenge thus arises, when we need to consider the two strains as metabolically distinct; each requiring different bioprocess conditions for optimal performances. The underperformance of the parental strain may have been exaggerated, if we have only tested conditions that intrinsically favoured the Δ*dxr* strain. Building on this work, one could systematically test across different conditions to obtain a broad comparison of their relative performance. In any case, our analyses were focused on the divergence in the strains’ evolutionary trajectories, which is indicative of trends over the longer term. We remain convinced that the evidence indicates the Δ*dxr* strain to be incapable of abolishing productivity, and that we have been successful with growth-coupling to restrict its evolutionary landscape.

### Growth-coupling to complement other strategies for engineering terpenoid bioproduction

Ultimately, this work is a proof of concept for a growth-coupling strategy in terpenoid bioproduction. Our approach to replace the native terpenoid biosynthetic pathway is broad in its scope and easy to implement, as has been demonstrated in studies employing other synthases and microbial chassis [77–79]. This suggests that the resultant growth-coupling—and our unique demonstration of its positive impact on bioproduction—can easily be transferred to other terpenoid bioproduction contexts as well. Moreover, our strategy easily complements the other strategies developed in the field, by acting in the background as a ‘fail-save’ against the fall in productivity below the imposed minimum. In this respect, we can further raise this limit by co-opting additional coupling mechanisms [15], and targeting the balance of reducing powers may be a viable candidate for the mevalonate pathway [80, 81]. All in all, we envision this strategy to cohere with other works in the enduring goal towards a commercially viable platform.

## Conclusion

To develop a commercially viable whole-cell biocatalytic platform, we need to address production heterogeneity across individual cells, and curtail any asymmetrical expansion of low and non-producing variants within the population. We designed a growth-coupling strategy, whereby a MEP-deficient *E. coli* chassis (Δ*dxr*) depended entirely on the heterologous mevalonate pathway for terpenoid biosynthesis. Here, the need to synthesise endogenous terpenoids imposes a minimum level of productivity for the target terpenoid, and prevents any inferior variants from propagating. Pitting the parental strain against the Δ*dxr* strain in a continuous bioproduction of linalool, we demonstrated the latter to have a better productivity profile in the first three days post-inoculation. Stability of linalool bioproduction across cell generations improved for the Δ*dxr* strain too. Crucially, the divergence in the evolutionary trajectory between the two strains was convincing that we were successful in growth-coupling. Functioning as a ‘fail-safe’ in the background of other strategies developed in the field, we envision our strategy to complement in the collective effort towards industrial terpenoid bioproduction.

## Supplementary Information

Supplementary material: Supplementary Figures 1-3.

## Data Availability

The datasets used and/or analysed during the current study are available from the corresponding author on reasonable request.
