# Supplementary material for: A growth-coupling strategy for improving the stability of terpenoid bioproduction in Escherichia coli
Source: Microb Cell Fact. 2024 Oct 16;23:279. doi: 10.1186/s12934-024-02548-1 (PMC11481808; doi:10.1186/s12934-024-02548-1)
Supplement: Supplementary file 1 — Supplementary material: Supplementary Figures 1-3. [file 12934_2024_2548_MOESM1_ESM.pdf]

SUPPLEMENTARY INFORMATION

A growth-coupling strategy for improving the stability of terpenoid bioproduction in *Escherichia coli*

Authors: Jing Chong Tan[1], Qitiao Hu[1], Nigel S. Scrutton[1] (corresponding author)

Affiliations:

[1] Manchester Institute of Biotechnology, The University of Manchester

131 Princess Street, Manchester, United Kingdom M1 7DN

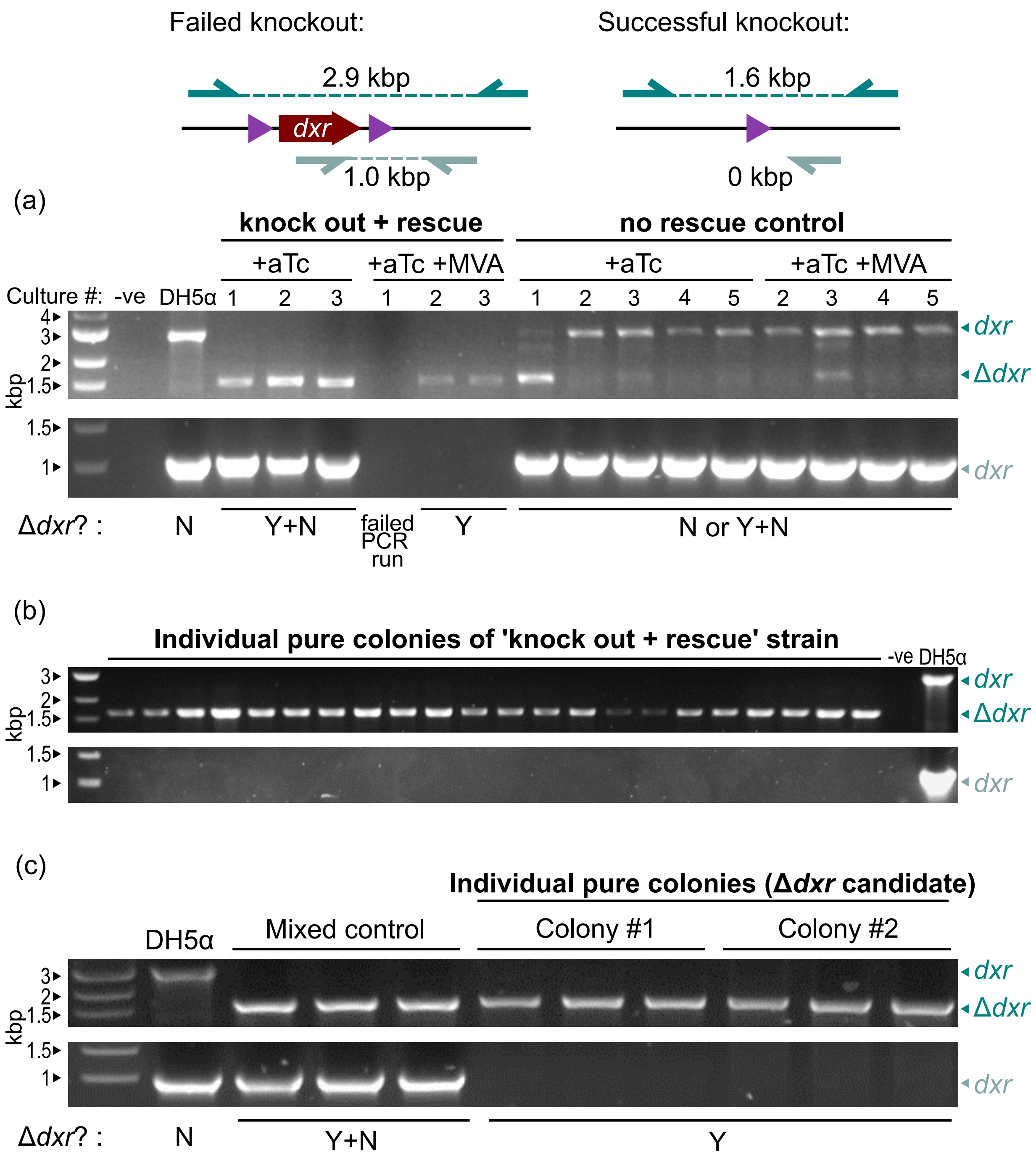

Figure S1: PCR verification for the status of the *dxr* locus.

PCR was performed with two sets of primers. The first (top dark teal, respective top gels) amplified across the *dxr* locus: an intact gene will produce a 2.9 kbp band, and a successful knockout will yield a 1.6 kbp band. The second (bottom light teal, respective bottom gels) amplified from within the locus: an intact gene will yield a 1.0 kbp band, and a successful knockout will yield no bands. The two PCR reactions can tally with each other to indicate an unsuccessful ('N') or successful ('Y') knockout. Conflicting results between the two PCR reactions indicates a mixed sample containing cells with and without the knockout ('Y+N').

The DNA ladder is on the leftmost lane. Where labelled, 'DH5α' strain represents a control for the intact *dxr* locus, and '-ve' represents a negative control for the PCR reactions.

(a) Selected cultures at the end of the assay in Figure 3(b) were analysed with colony PCR. Each lane represents an individual, specified culture ('#1-3/1-5'). Top labels indicates the strain, and the culture supplement(s) added. The results suggest that cells were able to escape the knockout induced by the Flp recombinase.

(b) At the end of the assay in Figure 3(b), the mixed cultures of the 'knock out + rescue' strain (grown with anhydrotetracycline and mevalonate) were streaked onto plates. Individual colonies were isolated and analysed with colony PCR. Each column represents an individual PCR reaction for an individual colony. The results of both primer sets concur that *dxr* was successfully knocked out for all colonies tested. Eleven colonies were then selected from this set for the growth assay in Figure 3(c).

(c) For the Δ*dxr* strain used in continuous linalool bioproduction, individual pure colonies were isolated as candidates. PCR was performed on genomic DNA extracted for verification of the knockout. 'Mixed control' represents the results obtained from a mixed population of cells with and without the knockout. For this control and the two candidates, each column represents one of three technical replicates of the PCR verification. The results of both primer sets concur that *dxr* was successfully knocked out for both candidates.

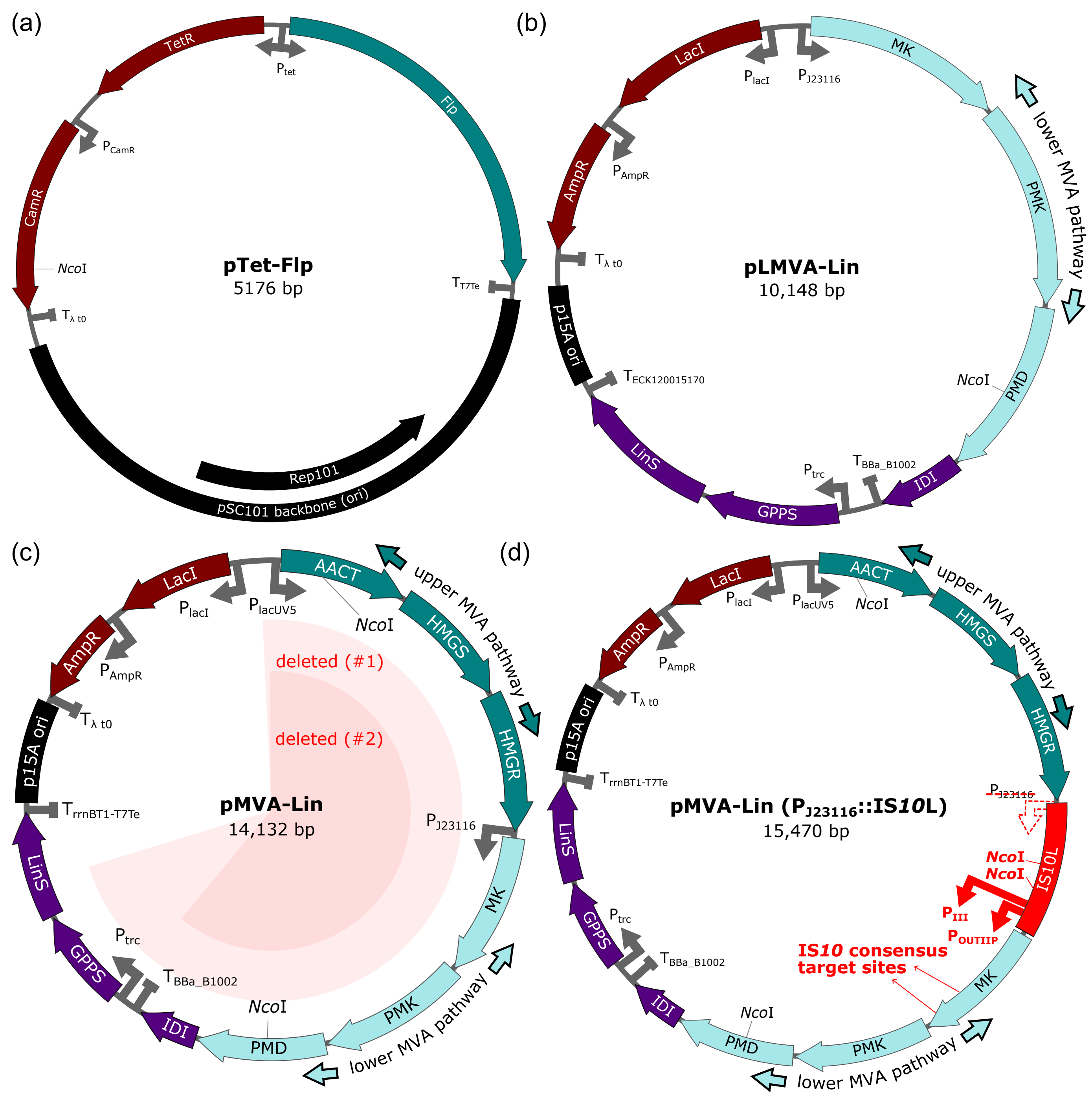

Figure S2: Maps of plasmids.

P and T: promoters and terminators respectively.
AmpR, CamR: enzyme for resistance to ampicillin/carbenicillin and chloramphenicol respectively.
ori: origin of replication.
TetR, LacI: Tet and Lac repressor respectively.
Flp: Flp recombinase.
IS10L: IS*10*-left.
Upper mevalonate (MVA) pathway:

AACT: acetoacetyl-CoA thiolase homologue from *E. coli*.
HMGS: 3-hydroxy-3-methylglutaryl-CoA synthase homologue from *Staphylococcus aureus*.
HMGR: 3-hydroxy-3-methylglutaryl-CoA reductase homologue from *S. aureus*.

Lower mevalonate (MVA) pathway:

MK: mevalonate 5-kinase homologue from *Saccharomyces cerevisiae*.
PMK: phosphomevalonate kinase homologue from *S. cerevisiae*.
PMD: diphosphomevalonate decarboxylase homologue from *S. cerevisiae*.

IDI: isopentenyl diphosphate delta-isomerase homologue from *E. coli*.
GPPS: (truncated) geranyl diphosphate synthase homologue from *Abies grandis*.
LinS: linalool synthase homologue from *Streptomyces clavuligerus*.

(a) Plasmid used for knocking out *dxr*. Anhydrotetracycline induces the expression of the Flp recombinase from P_{tet}. A single *Nco*I cut site linearises pTet-Flp into a 5.2 kbp fragment.

(b) Plasmid used for rescuing growth following the *dxr* knockout. The constitutively expressed lower mevalonate pathway converts supplemented mevalonate into the terpenoid precursor isopentenyl diphosphate (IPP). IDI is constitutively expressed to catalyse the conversion of IPP into the other precusor dimethylallyl diphosphate (DMAPP). IPP and DMAPP production restores endogenous terpenoid biosynthesis.

(c) Plasmid encoding the full mevalonate pathway for linalool bioproduction. IPTG induction of P_{lacUV5} expresses the upper mevalonate pathway to convert acetyl-CoA to mevalonate. The constitutive expression of the lower mevalonate pathway and IDI converts mevalonate to IPP and DMAPP. IPTG induces the expression of GPPS and LinS from P_{trc}. GPPS catalyses the condensation of one IPP to one DMAPP to form the geranyldiphosphate backbone, and LinS catalyses the latter's conversion into linalool. The two *Nco*I cut sites cleaves the plasmid into 7.6 and 6.5 kbp fragments.
The red sectors were deleted in the homologous-recombinant mutants of the parental strain (Run 2). The '#1' and '#2' deletion produces the 4.2 kbp and 5.6 kbp mutant plasmids respectively, with both retaining the determinants for replication (p15A ori) and antibiotic resistance (AmpR).

(d) The IS*10*-left inserted plasmid recovered from the Δ*dxr* strain. The insertion disrupted the constitutive P_{J23116} promoter, but expression of the operon may be rescued from the introduction of the promoters P_{III} and P_{OUTIIP}. The insertion introduced additional *Nco*I cut sites, which further cleaved the original 6.5 kbp fragment into 3.9, 3.7 and 0.3 kbp fragments. Only the first two were collectively observed as an additional single band. The insertion notably ignored its two consensus target sites found in the gene for MK.

Plasmid maps were created using the SnapGene® software.

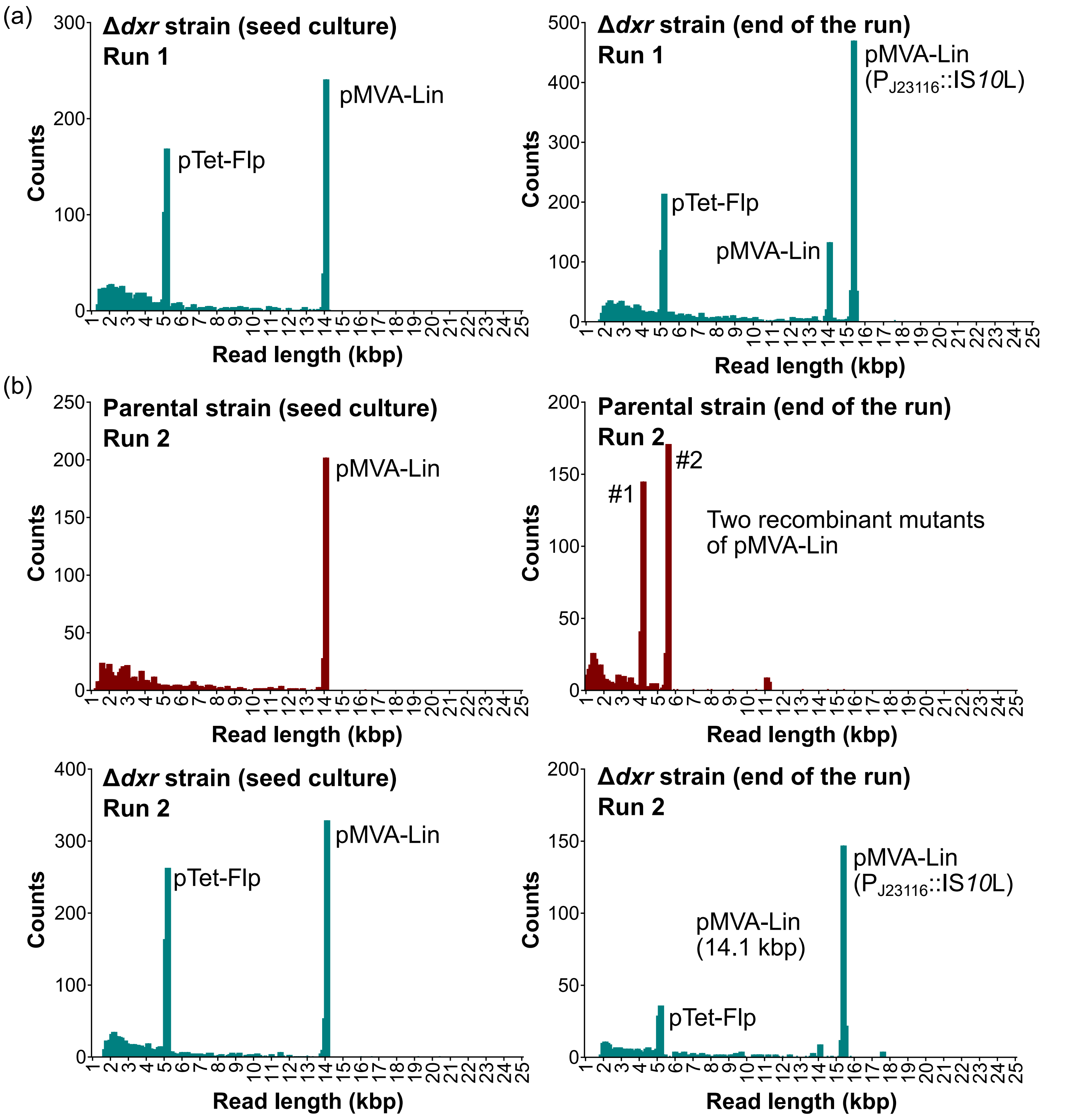

Figure S3: Frequency distribution of the sequencing read lengths.

Plasmids were extracted from the population and sequenced by Plasmidsaurus using Oxford Nanopore Technology. As plasmids were only linearised for processing, the read length should correspond to the plasmid size. The respective histogram chart thus gives a snapshot of the plasmid species present at the point of extraction. Each peak has been labelled with the expected plasmid according to its read length.

(a) Distribution for the Δ*dxr* strain in Run 1. Plasmids were extracted from the seed culture used for inoculating the reactor (left) and at the end of the run (right).

(b) Distribution for the parental (top row) and Δ*dxr* strain (bottom row) in Run 2. Plasmids were extracted from the seed culture used for inoculating the reactor (left column) and at the end of the run (right column).
Gel analysis suggested that the intact 14.1 kbp pMVA-Lin was still present in the Δ*dxr* population at the end of the run. The absence of a clear peak with counts above background levels suggests that its relative proportion in the population may be very low.
